# An Internal Ribosome Entry Site Directs Translation of the 3′-Gene from *Pelargonium Flower Break Virus* Genomic RNA: Implications for Infectivity

**DOI:** 10.1371/journal.pone.0022617

**Published:** 2011-07-26

**Authors:** Olga Fernández-Miragall, Carmen Hernández

**Affiliations:** Instituto de Biología Molecular y Celular de Plantas, Consejo Superior de Investigaciones Científicas-Universidad Politécnica de Valencia, Valencia, Spain; University of Cambridge, United Kingdom

## Abstract

*Pelargonium flower break virus* (PFBV, genus *Carmovirus*) has a single-stranded positive-sense genomic RNA (gRNA) which contains five ORFs. The two 5′-proximal ORFs encode the replicases, two internal ORFs encode movement proteins, and the 3′-proximal ORF encodes a polypeptide (p37) which plays a dual role as capsid protein and as suppressor of RNA silencing. Like other members of family *Tombusviridae*, carmoviruses express ORFs that are not 5′-proximal from subgenomic RNAs. However, in one case, corresponding to *Hisbiscus chlorotic ringspot virus*, it has been reported that the 3′-proximal gene can be translated from the gRNA through an internal ribosome entry site (IRES). Here we show that PFBV also holds an IRES that mediates production of p37 from the gRNA, raising the question of whether this translation strategy may be conserved in the genus. The PFBV IRES was functional both *in vitro* and *in vivo* and either in the viral context or when inserted into synthetic bicistronic constructs. Through deletion and mutagenesis studies we have found that the IRES is contained within a 80 nt segment and have identified some structural traits that influence IRES function. Interestingly, mutations that diminish IRES activity strongly reduced the infectivity of the virus while the progress of the infection was favoured by mutations potentiating such activity. These results support the biological significance of the IRES-driven p37 translation and suggest that production of the silencing suppressor from the gRNA might allow the virus to early counteract the defence response of the host, thus facilitating pathogen multiplication and spread.

## Introduction

Eukaryotic gene expression is highly controlled at translational level. The process of translation is composed by three phases, initiation, elongation and termination, of which initiation is considered to be the rate-limiting step and, consequently, it is the phase that is most often subjected to regulation [1], [2], [3]. Translational initiation usually takes place according to a mechanism of ribosome scanning that is 5′-end dependent and requires a 5′-cap and a 3′-polyadenylated tail [1], [4], two structures that are typically present in cytoplasmic cellular mRNAs. In the scanning mechanism, the 40S small ribosome subunit is recruited to the 5′-end of the mRNA, via a network of protein-protein and RNA-protein interactions, and undergoes a linear 5′ to 3′ migration until the first initiation codon (usually AUG) is reached. If this codon lies in an optimal sequence context, the subunit pauses, the translational initiation complex is formed and translation can proceed [5], [6]. Implicit in this model is that only the 5′-proximal gene in the mRNA is accessible to the ribosomes whereas other potential downstream genes are silent [2]. Just in some instances an mRNA whose translation is initiated by the scanning model may direct synthesis of more than one protein through mechanisms that modify ribosome behaviour as leaky scanning, reinitiation, frameshift or readthrough of leaky stop codons [7], [8], [9].

Although most mRNA species adheres strictly to the scanning model, a relatively small but growing number of mRNAs have been identified that are capable of initiating translation without the need for ribosome entry from the 5′ end. This alternative process involves the internal association of ribosome subunits at or near the initiation codon mediated by recognition of *cis*-acting motifs referred to as internal ribosome entry sites (IRESs). Such elements were first discovered in some animal viruses belonging to the family *Picornaviridae* [10], [11] and later on in members of other families like *Flaviviridae*, *Retroviridae*, *Herpesviridae* or *Dicistroviridae* (compiled in [12]). Evidence for internal initiation of translation has also been found in a number of plant viruses including some species of families *Potyviridae* [13], [14], [15], *Comoviridae* [16], *Tobamoviridae* [17], [18] and *Luteoviridae* [19]. Besides, the use of IRES elements as an alternate translation initiation mechanism operates in some cellular RNAs that can be expressed under conditions in which cap-dependent translation is impaired [20]. IRESs of distinct origin differ largely in structural organization, sequence, length and functional requirements [21]. The location of the IRES in the corresponding mRNA may also vary. Though they are usually found in long and highly structured 5′ untranslated regions (UTRs), IRESs at internal positions have also been reported, allowing polycistronic expression from a single mRNA [22].

*Pelargonium flower break virus* (PFBV) belongs to the genus *Carmovirus* in the family *Tombusviridae*. PFBV possesses a single-stranded plus-sense genomic RNA (gRNA) that is not capped at the 5′ end nor polyadenylated at the 3′ end. Such gRNA is 3,923 nt long and contains five open reading-frames (ORFs) flanked by untranslated regions of 32 and 236 nt at the 5′ and 3′ terminus, respectively [23]. The 5′-proximal ORF (ORF1) encodes a 27 kDa protein (p27) and terminates with an amber codon which may be readthrough to generate an 86 kDa protein enclosing the motifs conserved in the viral RNA dependent-RNA polymerases. Reverse genetics experiments have shown that both proteins are required for viral replication [24]. Two small ORFs, located in the central part of the viral genome, encode polypeptides of 7 (p7) and 12 kDa (p12), respectively, which are involved in virus movement [25] and the 3′-proximal ORF encodes a 37 kDa product (p37) which plays a dual role as coat protein (CP) and as suppressor of RNA silencing [26].

In accordance with the scanning mechanism that applies for the majority of eukaryotic RNAs, the PFBV 5′-ORFs encoding the replication proteins (p27 and its readthrough product p86) are likely translated from the gRNA. Conversely, the movement proteins (p7 and p12) and p37 must be translated later in the virus life cycle from two subgenomic RNAs (sgRNAs) of 1.7 and 1.4 kb, respectively, that are 3′-colinear with the gRNA and that are produced during PFBV infection [27]. Though this is the prevailing model for gene expression of carmoviruses (reviewed in [28]), protein p38 of *Hisbiscus chlorotic ringspot virus* (HCRSV, genus *Carmovirus*), encoded by the 3′-proximal gene and structurally and functionally homologous to PFBV p37, has been found to be translated not only from a sgRNA but also from the viral gRNA through an IRES that has been mapped immediately upstream of the p38 coding sequence [29]. Here we show that, as HCRSV, PFBV gRNA contains an IRES that mediates translation of p37, thus suggesting that this expression strategy might be common in members of the genus *Carmovirus*. Through deletion and mutagenesis studies, we have determined some structural prerequisites of the IRES element of PFBV which seem to be partly different from those reported for the IRES of HCRSV. The function of PFBV IRES has been proven both *in vitro* and *in vivo* and, most remarkably, we provide evidence on the relevance of this element for virus infection.

## Results

### PFBV gRNA directs synthesis of p27, p86 and p37

**Figure 1:**
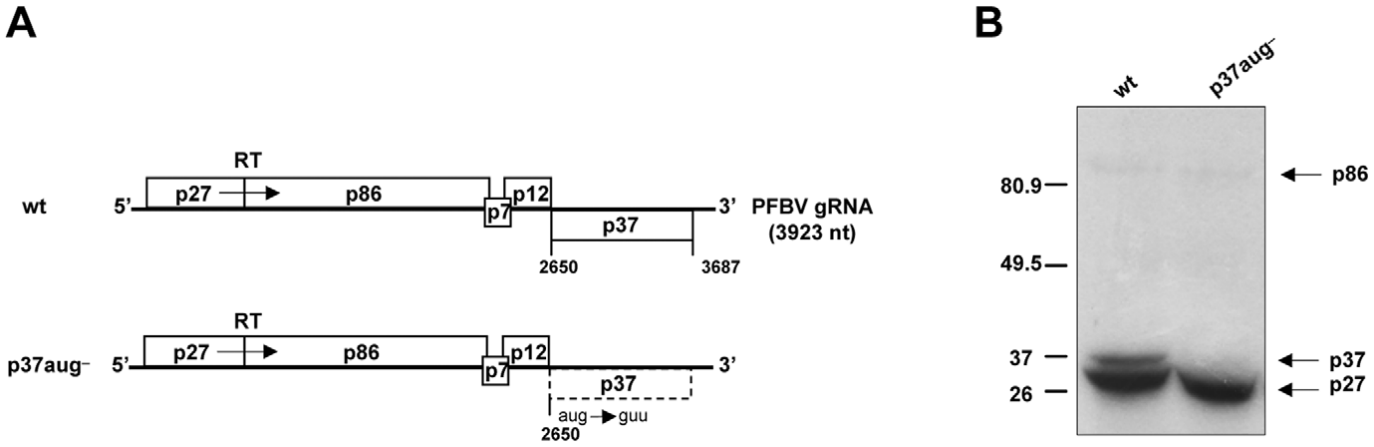
Analysis of translation products from PFBV gRNA. (A) Schematic representation of PFBV genome organization (derived from wt construct pSP18-IC) and of mutant p37aug-. Open boxes represent ORFs and numbers correspond to nucleotide positions defining each ORF. Nucleotide substitutions in mutant p37aug- are indicated. In this mutant, the dashed box denotes the ORF that is not expected to be translated. RT: readthrough of the ORF(p27) stop codon. (B) *In vitro* translation assay with WGE of PFBV transcripts derived from the wt and p37aug- constructs. Translation products labeled with [^14^C]leucine were separated on SDS-12% polyacrylamide gels and visualized by autoradiography. Positions of protein molecular weight markers (in kDa) are depicted on the left side of the autoradiograph and PFBV proteins are indicated on the right side.

To test whether PFBV gRNA serves as mRNA for translation of the two 5′-proximal ORFs as expected, wild-type (wt) transcripts derived from the infectious clone of the virus pSP18-IC [30] were subjected to *in vitro* translation reactions with wheat germ extracts (WGE). Separation of the translation products by SDS-PAGE confirmed the expression of the replication proteins, p27 and p86 (Figure 1). However, an additional product with a size consistent with that of p37, encoded by the 3′-proximal ORF, was also detected. Mutation of ^2650^AUG, the start codon of ORF(p37), to GUU in construct p37aug-, abolished the synthesis of the latter product thus confirming that PFBV gRNA directs translation of p27, p86 and p37 (Figure 1).

The possibility that p37 would be expressed from a monocistronic degradation product of the gRNA instead of the full-length molecule was explored by analyzing the integrity of the translation template during *in vitro* translation reactions. Northern blot analysis with a PFBV-specific probe of RNA extracted from aliquots of the translation reaction mixture taken at regular intervals, did not reveal the presence of any outstanding degradation product (data not shown) indicating that synthesis of p37 from the gRNA did not result from an endonucleotytic cleavage that led to a shorter RNA. Other mechanism(s) should be responsible for synthesis of p37 and an internal initiation event was considered as the most plausible one.

### An IRES element mediates expression of p37 from PFBV gRNA

**Figure 2:**
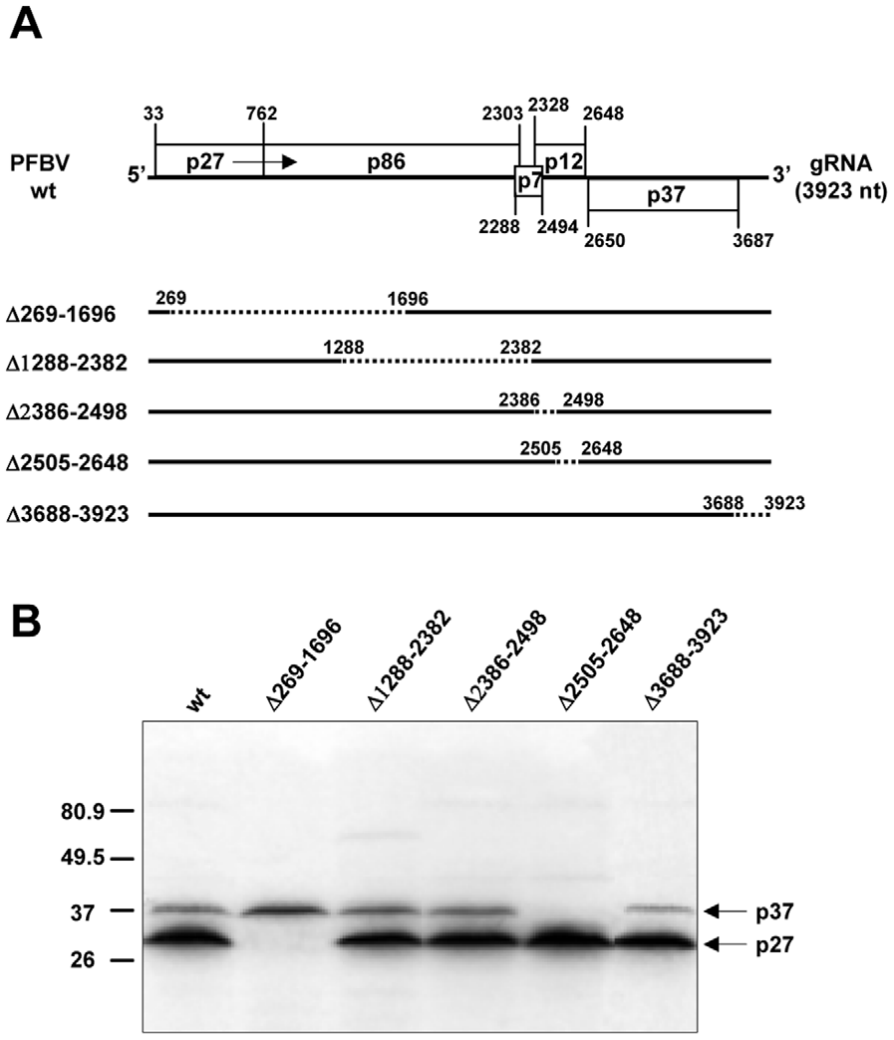
Effect of deletions on the translation pattern of PFBV gRNA. (A) Schematic representation of the PFBV wt and deletion mutants. Deleted regions are represented by dashed lines and the borders of the deletions are specified. (B) *In vitro* translation assay with WGE of PFBV transcripts derived from the wt and mutant constructs as indicated above the lanes. The band above the 80.9 kDa weight marker corresponds to the p86 replicase and is present in those mutants that do not compromise the readthrough mechanism; other intermediate bands are quimeric proteins resulting from the deletion introduced in some of the mutants. Other details as in Fig. 1.

In order to assess whether an IRES could be directing translation of p37 from the PFBV gRNA, a deletion analysis was carried out to delineate the region responsible for putative internal initiation. Deletion of regions embracing nt 269–1696, 1288–2382, 2386–2498 and 3688–3923 did not significantly affect p37 production (Figure 2). However when the segment comprised between nt 2505 to 2648 was removed, synthesis of p37 was abolished pointing to the presence of an IRES in the deleted region.

**Figure 3:**
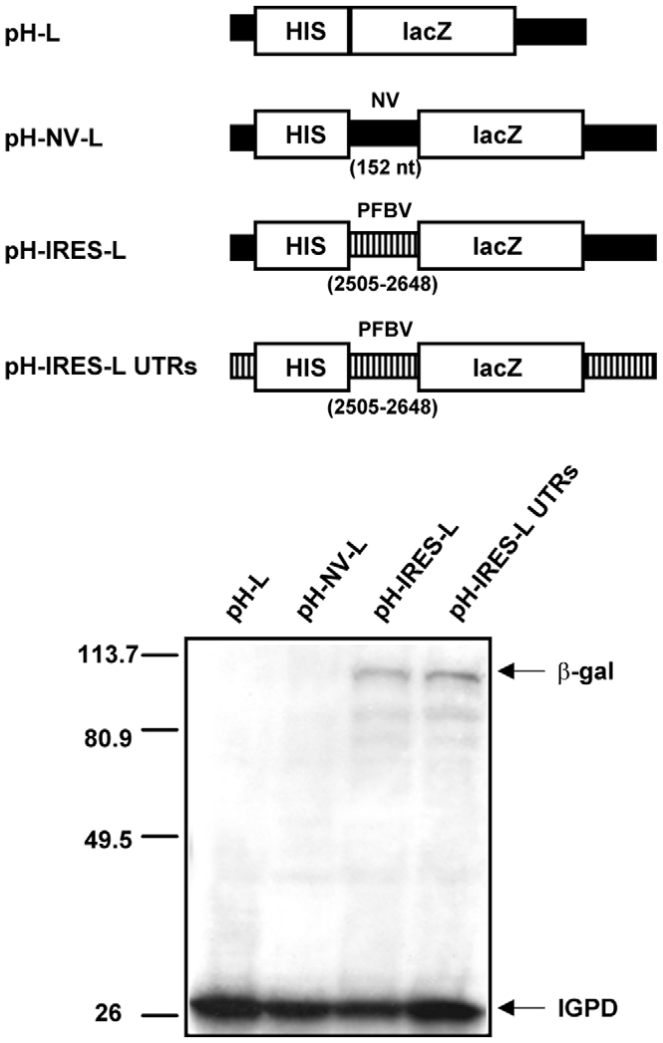
Translation assays with bicistronic constructs in WGE. Schematic representation (top) and analysis of *in vitro* translation products (bottom) from linearized transcripts of HIS3/lacZ bicistronic constructs. In top panels, solid and stripped lines represent non-PFBV and PFBV sequences, respectively. The size of the non-viral sequence (NV) and the positions in the viral genome of the PFBV segment inserted between the cistrons are indicated within brackets. In bottom panels, positions of the HIS3 and lacZ translation products (IGPD and ß-gal, respectively) are marked on the right side. Other details as in Fig. 1.

IRESs have been proven to drive protein production from the second ORF of bicistronic mRNAs when placed outside of its genetic context [11], [31], [32]. To further confirm the IRES function of the 143 nt delineated segment, this genomic portion was inserted into a bicistronic construct containing the HIS3 gene as the 5′ cistron and the lacZ gene as the 3′ cistron giving rise to pH-IRES-L. Construct pH-L, with no inserted sequences between the 5′ and 3′ cistrons, and construct pH-NV-L, containing a 152 nt non-PFBV DNA between the HIS and lacZ genes, were used as negative controls of IRES activity. *In vitro* translation assays with the bicistronic constructs resulted in the detection of HIS3 encoded-product (imidazole glycerol-phosphate dehydratase, IGPD) in all cases but expression of the lacZ gene, the 3′ cistron, was only observed with the construct containing the selected PFBV region between the 5′ and 3′ cistrons (Figure 3). Thus the results indicated that translation of ß-galactosidase was initiated by the inserted sequence.

Activity of some viral IRESs has been reported to be enhanced by the corresponding UTRs [33], [34]. Deletion of the PFBV 3′ UTR in construct Δ3688–3923 did not lead to any significant change in p37 production (Figure 2), indicating that there is no synergism between the PFBV IRES and the 3′ UTR. To test further whether the viral UTRs have any effect on IRES function, the terminal regions of the bicistronic construct pH-IRES-L were replaced by those of PFBV to generate construct pH-IRES-L UTRs. The corresponding *in vitro* translation assays showed that the addition of the PFBV UTRs might not modify the production level of ß-galactosidase (Figure 3), thus confirming that observed in the viral context. The results corroborated the IRES condition of the PFBV internal region and suggested that its activity was not influenced by the viral UTRs.

### Assessment of length and structural requirements of the PFBV IRES

**Figure 4:**
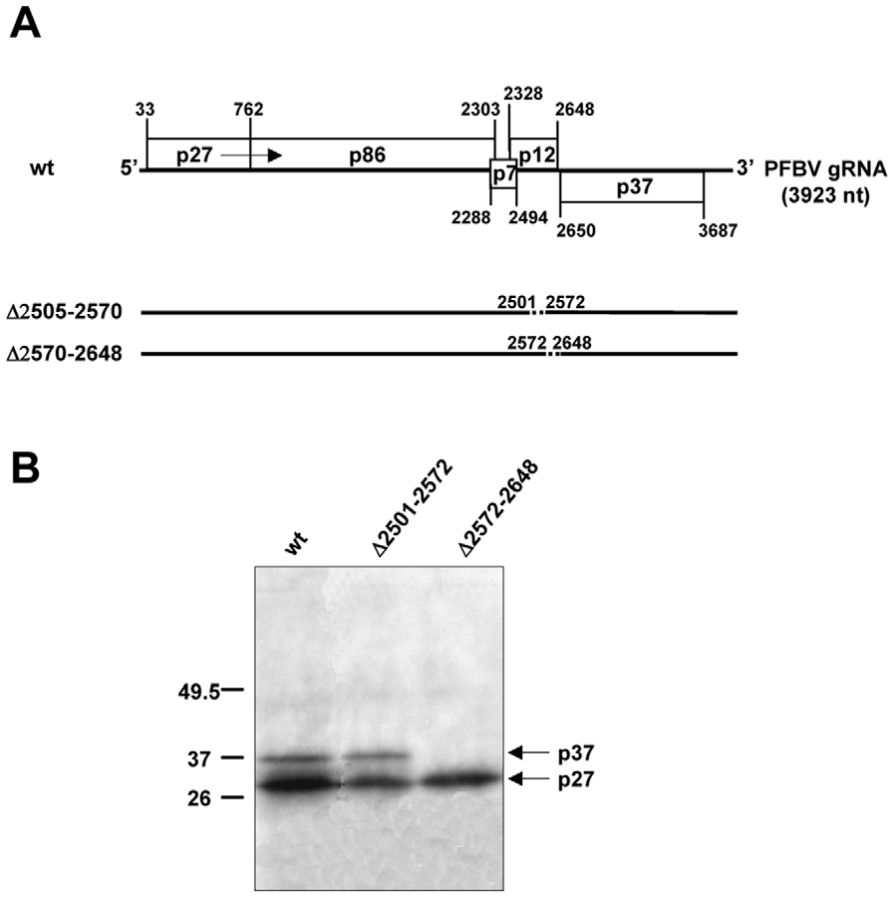
Further delineation of PFBV IRES. (A) Schematic representation of wt PFBV and mutants thereof carrying deletions. In the mutants, deletions are represented by dashed lines. (B) *In vitro* translation assay with WGE of PFBV transcripts derived from the wt and mutant constructs. Other details as in Fig. 1.

*In silico* analysis of the 143 nt region delineated above indicated that it may fold into a hairpin-like structure (data not shown). However, the free energy of such structure was relatively high (ΔG = −20.7) suggesting that it could be functionally irrelevant. In order to confirm this point and to map the IRES element more precisely, two additional mutants were generated by deleting either the 5′ side (Δ2501–2572; Figure 4) or the 3′ side (Δ2572–2648; Figure 4) of the predicted hairpin. Whereas removal of nt 2501–2572 had a minimal effect on p37 production when compared with the wt construct, elimination of nt 2572–2648 abolished p37 synthesis, indicating that the latter segment, with a length of about 80 nt, contained the active IRES element.

**Figure 5:**
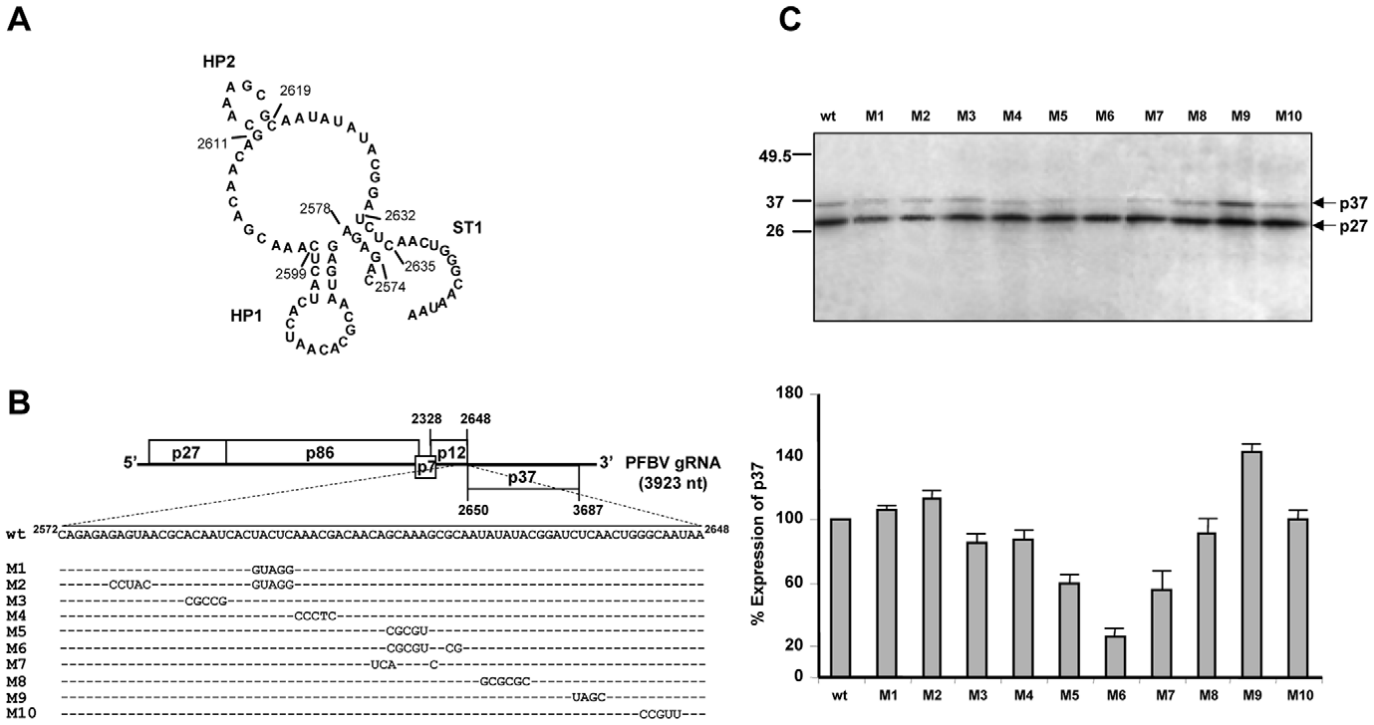
Analysis of structural requirements of the PFBV IRES. (A) *In silico* folding of PFBV IRES (encompassing nt 2572–2648) showing the predicted hairpin (HP1 and HP2) and stem (ST) structures. (B) Schematic representation of wt PFBV and mutants thereof carrying nucleotide substitutions in the IRES region. Dots indicate residues identical to the wt sequence. (C) Autoradiograph showing the results of *in vitro* translation assays with WGE using PFBV transcripts derived from the wt and mutant constructs. A graphic representation of production levels of p37 is shown underneath. The expression of p37 from wt transcripts is represented as 100% and the effects of mutations on the translation of ORF(p37) from other templates are represented as percentages of the p37 expression from the wt. Other details as in Fig. 1.

Computer predictions with the region embracing the IRES revealed that it was relatively unstructured with only two small hairpins located between nt 2578–2599 (HP1) and 2611–2619 (HP2), and a small stem (ST) formed by pairing between nt 2574–2577 and nt 2632–2635 (Figure 5). Nucleotide substitutions disrupting the stem of HP1 (change ^2595^ UACUC to GUAGG) in mutant M1 did not significantly affect p37 production that remained to essentially wt levels (Figure 5). Introduction of compensatory mutations (^2578^ GAGUA to CCUAC) in mutant M2 to restore the putative HP1 or alteration of the sequence of the terminal loop of HP1 (^2587^ ACAAU to CGCCG) in mutant M3 only caused a slight reduction in p37 production, indicating that the primary and/or secondary structure of the region embracing HP1 has little influence on IRES activity (Figure 5). Nucleotide changes affecting the 5′ side of HP2 (^2611^ GCAAA to CGCGU) in mutant M5 diminished p37 production to 65%, considering 100% production that of the wt (Figure 5). Introduction of compensatory mutations (^2618^ GC to CG) in mutant M6 to restore the stem of HP2 diminished p37 production further (to 25%, Figure 5), suggesting that primary structure rather than secondary structure of the altered segment was relevant for IRES function. Consistently with this results, change of ^2610^ AGC and ^2618^ G to UCA and C, respectively, in mutant M7 also led to a substantial reduction in p37 synthesis (to 60%, Figure 5), underscoring the importance of the targeted region. When the ST was disrupted by converting sequence ^2633^ CUCA to UAGC in mutant M9, p37 production was raised to 140% pointing to another critical primary/secondary structure in the IRES. Finally, mutation of predicted single-stranded regions in constructs M4 (^2600^ AAACG to CCCTC), M8 (^2622^ UAUAUA to GCGCGC) and M10 (^2641^ GGCAA to CCGUU) had negligible effects on production levels of p37 (Figure 5). Summarizing, two regions, involved in potential formation of HP2 and ST, appeared to play a key role within the IRES.

### Differential involvement of the IRES element on p37 expression from sgRNAs

**Figure 6:**
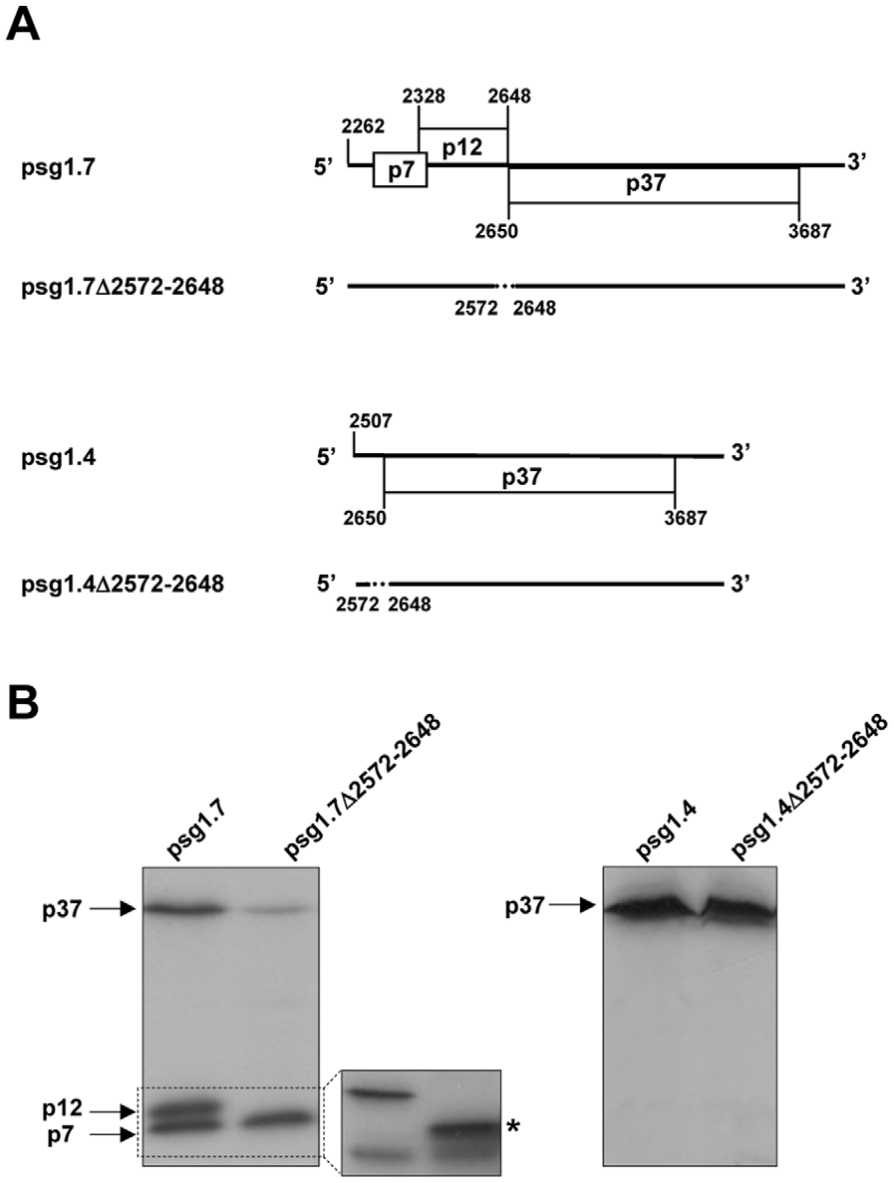
Assessment of IRES function from PFBV sgRNAs. (A) Schematic representation of the 1.7 kb and 1.4 kb sgRNAs (whose cDNAs have been cloned in constructs psg1.7 and psg1.4, respectively) and of deletion mutants derived from them. Deleted regions are represented as dashed lines. Numbers correspond to nucleotide positions in the gRNA. (B) *In vitro* translation assay with WGE of PFBV transcripts derived from the wt and mutant sgRNA constructs. Translation products labeled with [^14^C]leucine were separated on SDS-15% polyacrylamide gels and visualized by autoradiography. In the left panel, resolution of the smaller proteins in a SDS-20% polyacrylamide gel is shown with an asterisk marking the position of a product of 10 kDa that is synthesized as a result of the segment removed in mutant psg1.7Δ2570–2650.

To study if the IRES element could be functioning in PFBV sgRNAs, transcripts derived from constructs psg1.7 and psg1.4, containing sequences corresponding to the 1.7 kb and 1.4 kb sgRNAs, respectively, were subjected to *in vitro* translation reactions with WGE (Figure 6). Three proteins with apparent molecular masses of 7, 12 and 37 kDa were synthesized from psg1.7 transcripts indicating that the 1.7 kb sgRNA is not only structurally but also functionally tricistronic and directs synthesis of the two movement proteins (p7 and p12) and of p37. As expected, only p37 was produced by *in vitro* translation of psg1.4 transcripts. Remarkably, deletion of the IRES-containing segment in psg1.7Δ2572–2648 strongly reduced the expression of p37. Conversely, when the same deletion was introduced in the 1.4 kb sgRNA-derived construct psg1.4Δ2572–2648, no alteration in p37 production was observed. Overall, the results indicated that the IRES element is functionally active in the 1.7 kb sgRNA but not in the 1.4 kb sgRNA.

### Analysis of the PFBV IRES function *in vivo*

**Figure 7:**
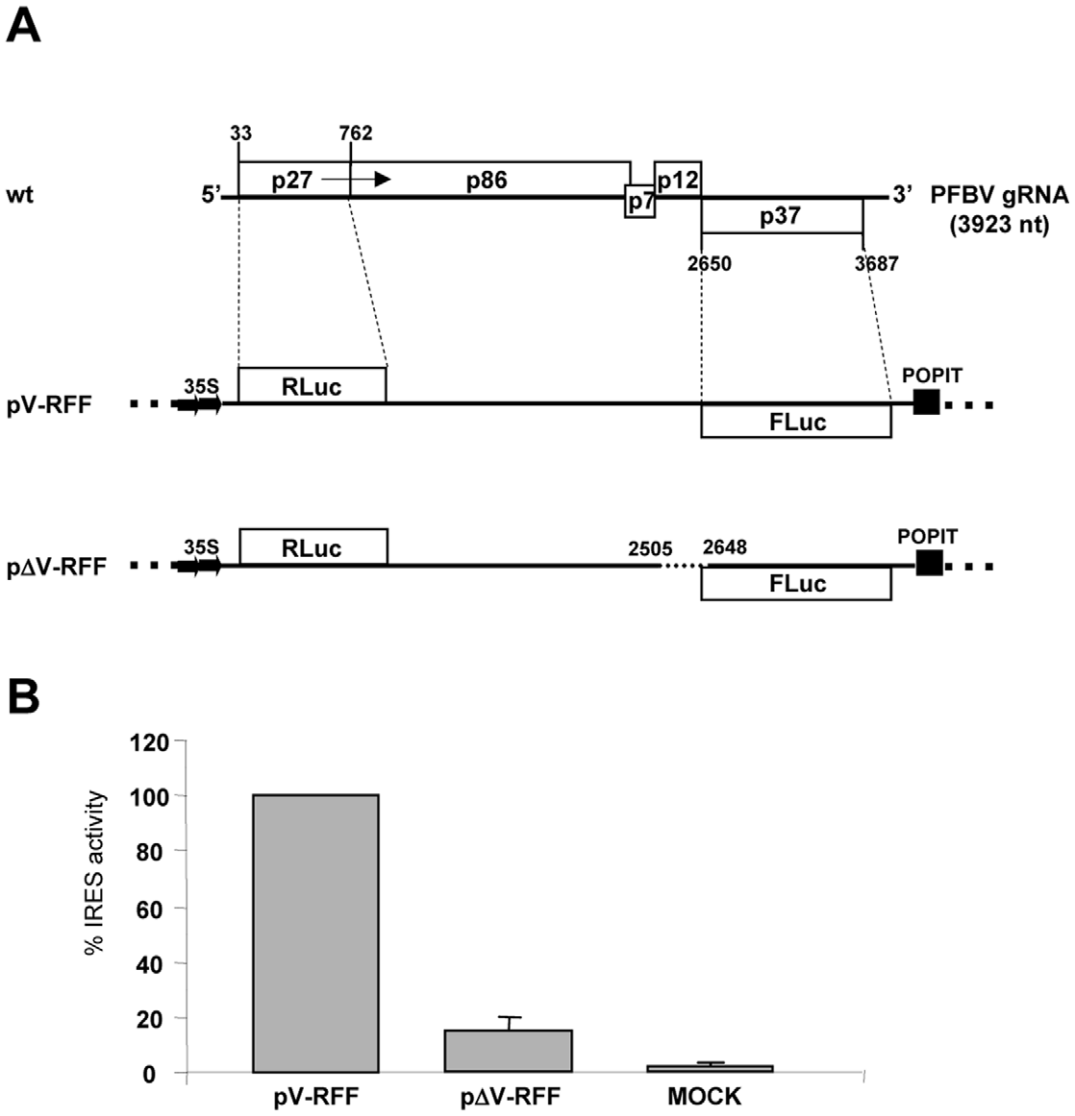
Evaluation of internal initiation of translation in plant cells. (A) Schematic representation of constructs pV-RFF and pΔV-RFF used for agroinfiltration assays. Rluc and Fluc genes are shown as open boxes. Solid lines represent PFBV sequences with numbers above indicating positions in the viral genome. The deleted segment in construct pΔV-RFF is depicted by a dashed line. (B) Fluc activities measured in *N. benthamiana* leaves agroinfiltrated with constructs pV-RFF and pΔV-RFF. Mock agroinfiltrated leaves were included as controls. Rluc activity was used to normalize Fluc activity in each assay. Fluc activity from the pV-RFF construct was set as 100%. Average of four independent experiments is shown.

In addition to the *in vitro* experiments, the functionality of the IRES was studied *in vivo* through *Agrobacterium*-mediated transient expression assays. To this aim, ORFs p27 and p37 in PFBV genome were replaced by the *Renilla* luciferase (Rluc) and *Firefly* luciferase (Fluc) genes, respectively. The resulting chimeric viral cDNA was inserted between the CaMV 35S promoter and the terminator sequence of the *Solanum tuberosum* proteinase inhibitor II gene (PoPit) and cloned into a binary vector to yield plasmid pV-RFF. An equivalent construct, named pΔV-RFF, with an internal deletion comprising the IRES was also generated (Figure 7, upper panel). Rluc and Fluc activities were readily detected in *Nicotiana benthamiana* leaves patches agroinfiltrated with construct pV-RFF providing evidence for internal initiation of translation promoted *in vivo* by a central segment of PFBV genome. Moreover, Fluc expression was 10-fold reduced in the case of pV-ΔRFF indicating that the region functioning as IRES in plant cells may correspond essentially to that determined in wheat germ cell-free extracts (Figure 7, lower panel).

### Mutations that affect IRES activity led to alterations in viral infectivity

**Figure 8:**
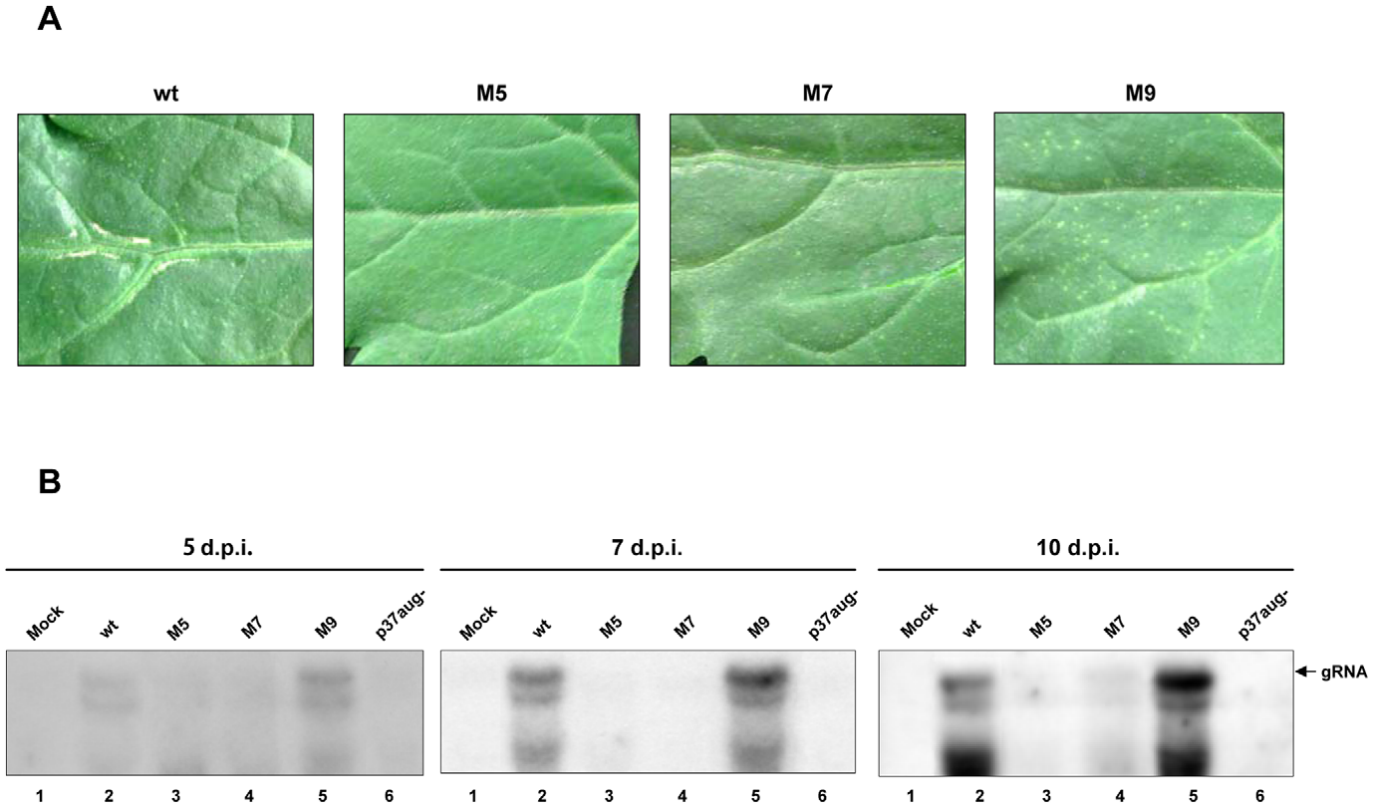
Effects of mutations affecting IRES function on virus infectivity. (A) Symptoms on *C. quinoa* leaves inoculated with wt construct pSP18-IC or with mutants M5, M7 and M9. Pictures were taken at 7 d.p.i. (B) Northern blot analysis of leaves inoculated with the above constructs and with mutant p37aug- (included as negative control). Total RNA samples were run in agarose gel, transferred to nylon membranes and hybridized with a probe derived from the 3′-terminal region of PFBV. Leaves were collected at 5, 7 and 10 d.p.i. as indicated above the blots. The arrow marks the position of PFBV gRNA; lower bands correspond to sgRNAs.

The experiments described above substantiated the *in vivo* functionality of the PFBV IRES in a viral sequence context. In order to assess whether the identified element plays actually a role during PFBV infection, mutants M5 and M7, in which the IRES activity was reduced to 59 and 56%, respectively, in *in vitro* translation assays (Figure 5), and mutant M9, in which the IRES activity was increased to 143% (Figure 5), were bioassayed in *Chenopodium quinoa*. The nucleotide substitutions introduced in these mutants, though affected IRES function, did not alter the amino acid sequence encoded by the overlapping ORF(p12) except in the case of M5 in which the corresponding p12 would present two conservative amino acid changes, S95T and K96R. The mutant in which the start codon of the p37 gene had been abolished (p37aug-, Figure 1), was also included. Mutant M6, showing the lowest IRES activity (Figure 5), was not considered because the corresponding nucleotide replacements led to non-conservative amino acid changes in p12. The number of lesions induced by mutants M5 and M7 was very much reduced (more than 20-fold) and their appearance delayed (at least two days) when compared with the wt construct. Conversely, mutant M9 induced lesions that came earlier (about two days) and exceeded in number those induced by the wt transcripts. The lack of lesions on leaves inoculated with mutant p37aug-, in which the production of p37 was completely impaired, confirmed that the protein is strictly required for effective infection. Northern blot hybridization of the inoculated tissue corroborated visual inspections: no PFBV was detected in p37aug- inoculated leaves at any time post-inoculation whereas accumulation of viral RNAs in M5-/M7- and M9-inoculated leaves was lower and higher, respectively, than that found in leaves inoculated with the wt construct (Figure 8). These results strongly suggested that p37 translation is mediated *in vivo* by the IRES element and that unbalanced production of the protein results in remarkable effects on viral infectivity.

## Discussion

In this study, we provide evidence that the 3′-proximal p37 gene of PFBV is expressed from the gRNA through an IRES. This element is located immediately upstream of the p37 gene and deletion analyses and *in vitro* translation assays have allowed to conclude that it is contained within a 80 nt region that lacks extensive secondary structure. These traits resemble those of HCRSV IRES [29] despite both elements do not share significant sequence similarity. Structural simplicity seems to be a hallmark not only of carmoviral IRESs but of IRESs from plant viruses that are considerably short and devoid of strong secondary structure(s) [22] as opposed to IRESs from a animal viruses that have lengths ranging from 200–450 nt and adopt complex conformations [35], [36], [37].

Study of structural requirements of PFBV IRES has revealed that the segment encompassing nt 2611–2619 and potentially involved in formation of a hairpin (HP2), might be relevant for IRES function. The adoption of the predicted hairpin did not apparently contribute to IRES activity as mutations affecting this region reduced p37 production at considerable levels, irrespective of whether they preserved the proposed folding or not (mutants M5–M7 in Figure 5). It is worth noting the presence of a GCAA sequence, compatible with a GNRA motif, among nt 2611–2614 that was altered in all cases by the introduced mutations. The functional relevance of this type of motifs has been demonstrated in picornavirus IRESs [38], [39], [40] though they appear in a tetraloop conformation in contrast to that predicted for the GNRA-like motif of PFBV IRES. Another critical region for IRES activity was identified among nt 2632–2635 which is potentially implicated in formation of a stem (ST), though the importance of the secondary structure element rather than the nucleotide sequence has not been proven. Interestingly, the results of *in vitro* translation with the mutant that targeted the latter region (M9, Figure 5), suggested that the IRES function might be optimized, as p37 production was increased by 1.5-fold with respect to the wt. The noticeable effects on the efficiency of PFBV IRES observed after modification of a low number of nucleotides parallel results obtained with other IRES elements showing severe reductions in IRES activity caused by single nucleotide substitutions [38], [39], [40], [41]. It should be also noted that the sequence of the PFBV IRES is considerably biased to A residues (42.5%), as found for the IRES element of crucifer infecting tobamovirus where polypurine (A)-rich sequences have been shown to be crucial for promoting internal ribosome entry in different systems [42]. However, alteration of A-rich tracts of PFBV IRES in mutants M4, M8 or M10 had negligible effects on p37 production (Figure 5) suggesting that this type of stretches play merely a conformational role by reducing secondary structure and, therefore, by providing a suitable landing pad for ribosomes to initiate at the downstream initiation codon. A similar role has been proposed for a CA-rich region of HCRSV IRES [29] and for unstructured RNA segments of other eukaryotic IRESs [43].

*In vitro* assays with bicistronic constructs have revealed that PFBV IRES is not only active in its natural, viral context, but also in heterologous context(s) in agreement with that reported for numerous IRESs [10], [11], [31], [32]. In addition, we have corroborated IRES activity *in vivo* through replacement of the 5′- and 3′-proximal viral ORFs by reporter genes that allowed quantification of translation either dependent of the 5′-end or promoted by an internal region. These experiments supported that the PFBV segment endowed with IRES activity *in vivo* corresponds essentially to that delineated *in vitro*, though we cannot discard that requirements for internal initiation of translation in plant cells may differ at least in part from requirements in a cell-free translation system. Small variations in structural demands for IRES activity *in vivo* and *in vitro* have been previously reported [19], [44], [45] likely conditioned by conformational alterations and/or by differences in assisting protein factors. Additional work will allow further characterization of structure-function relationships in PFBV IRES both *in vitro* and *in vivo*.

Conversely to that reported for the IRES of HCRSV [30] and for other IRES elements [33], [34], [46], [47], [48], [49], no appreciable effect of the viral 3′ UTR on the efficiency of PFBV IRES-mediated translation was observed from neither studies with the gRNA nor the bicistronic constructs, at least *in vitro*. The apparent co-operation among the HCRSV IRES, located as that of PFBV in a central portion of the genome, and the 3′ UTR for translational enhancement was referred to as an exception as so far all IRESs reported to have synergy with either the 3′ UTR or the poly(A) tail are found in the 5′ UTR [29]. The lack of cross-talk among the PFBV IRES and the corresponding 3′ UTR suggests mechanistic differences with respect to HCRSV IRES. It is also worth noting that no clear reduction in the expresion of the PFBV 5′ ORFs (p27 and p86) was observed after deletion of the 3′ UTR suggesting that comunication among the 5′ and 3′ termini of the viral RNA is not essential for efficient translation from the 5′ end. These results differ from those reported for HCRSV, *Turnip crinkle virus* and *Melon necrotic spot virus*, all carmoviruses, showing the presence of translational enhancers (TEs) in their corresponding 3′ UTRs [50], [51], [52] as described in other plant viruses lacking 5′-cap structure (reviewed in [53]). These TEs are proposed to boost 5′ end-dependent translation in the absence of cap through pseudo-circularization of mRNAs and recruitment of components of the translational machinery [51], [54]. Some of the TEs are, however, not responsive in traditional *in vitro* assays [51], [55] and thus we cannot exclude the possibility that a potential PFBV TE has been overlooked with our experimental approach.

Further results of the present study indicate that the PFBV IRES is active from the gRNA and also from the 1.7 kb sgRNA but not from the 1.4 kb sgRNA. The latter observation contrasts with that reported for the corresponding sgRNA of HCRSV [56] and suggests that a 5′-dependent scanning-like mechanism accounts for p37 translation from the smallest, monocystronic PFBV sgRNA. It is likely that the active conformation of the IRES is not achieved in the context of this sgRNA or, alternatively, it could be outcompeted by efficient ribosome scanning from the 5′ end. On the other hand, it should be noted that IRES deletion abolished p37 production from the gRNA whereas such deletion reduced but not completely eliminated p37 expression from the 1.7 kb sgRNA (Figure 6), suggesting the contribution of other mechanism(s) in the latter case. A plausible possibility would be a leaky scanning event similar to that described recently for CP translation from the single sgRNA of Pelargonium line pattern virus [57]. Coexisting strategies have also been suggested for expression of the movement protein of *Tobacco mosaic virus* from I2 sgRNA that could be mediated both by ribosome scanning and by an IRES [18]. In any case, despite p37 can be produced from sgRNAs by different mechanisms, the IRES-mediated translation of the protein from the gRNA at the very early stages of infection might provide an advantage to the virus. This advantage could derive from the RNA silencing suppressor activity of p37 that would be critical to counteract one of the main defence responses of the host, thus facilitating multiplication and spread of the pathogen. In agreement with this view, mutations that diminish the IRES function strongly reduced the infectivity of the virus while the progress of the infection was favoured by mutations that potentiate IRES activity. Such direct correlation nicely supports and underscores the importance of the IRES-driven p37 expression during the virus life cycle.

To conclude, the finding of an IRES that directs expression of the 3′-proximal gene from PFBV gRNA as reported previously for HCRSV, raises the question as to whether all carmoviruses employ a similar translation strategy. Nucleotide sequence comparison of the 150 nt regions upstream CP genes reveal they are not highly conserved among carmoviruses (data not shown); however, this should not be taken as an argument against the existence of a common strategy of IRES-mediated translation in this viral group since IRES elements may be well differentiated in nucleotide sequence and RNA structure even among related viruses [36], [58]. Another challenging question concerns to the identification of the mechanism for ribosome recruitment by the IRES. Most likely, such recruitment is mediated by association of the IRES to protein factors that may act as chaperones and/or establish bridges with the ribosomes [59], [60]. Alternatively, direct base pairing with the 18S rRNA could guide ribosome engagement as suggested for different IRES including that of HCRSV [29], [61], [62] though this possibility has only been formally demonstrated in one case [63]. A search for sequence complementary among PFBV IRES and the 18S rRNA has yielded numerous stretches in which base pairing may occur (data not shown) though we could not establish a clear link among the potential base pairing(s) and mutations that affect IRES function. Identification of protein factors to which PFBV IRES can bind will undoubtedly provide important clues on the regulation of the translation mediated by this viral element.

## Materials and Methods

### Plasmid construction and mutagenesis

Plasmid pSP18-IC, which contains a wt full-length PFBV cDNA inserted into pUC18 downstream from a T7 RNA polymerase promoter [30], was used as template to generate a set of mutant constructs bearing deletions or nucleotide substitutions. To delete some internal regions in the viral sequence in constructs pPFBVΔ269–1696, pPFBVΔ1288–2382 and pPFBVΔ2386–2498, plasmid pSP18-IC was digested with the restriction enzymes *Xho*I/*Sal*I, *Eco*RV, and *Eco*RV/*Sph*I, respectively, which have targets within the viral cDNA. The digestion products were separated in agarose gels and the proper DNA band was eluted and religated either directly (bands with compatible ends) or after end-polishment with T4 DNA polymerase (band with incompatible ends). To generate four additional deletion mutants, pPFBVΔ2505–2648, pPFBVΔ2501–2572 and pPFBVΔ2572–2648, plasmid pSP18-IC was used as template for PCR reactions with *Pfu Turbo* DNA polymerase (Stratagene) and different pairs of 5′-phosphorilated primers that were complementary and homologous to distinct regions of the PFBV sequence. PCR products (comprising the cloning vector, pUC18, fused to a PFBV 5′ region at one side and to a PFBV 3′ region at the other side) were eluted after agarose electrophoresis, self-ligated and used for transformation. A viral cDNA lacking the complete 3′ UTR was amplified from plasmid pSP18-IC using the Expand High Fidelity PCR System (Roche) and primers CH61 (5′-GCAAGCTTG*TAATACGACTCACTATAGGG*AACAAAATGGCACACTATTTTGG-3′), which contains a *Hin*dIII site (underlined) fused to a T7 RNA polymerase promoter sequence (in italics) followed by 23 nt of the 5′ end of the virus sequence, and CH100 (5′-CCCTGCAGTCATAGCCTAGATACGTACCAC-3′), complementary to nt 3666–3687 of the PFBV gRNA with an *Pst*I site (underlined) at the 5′ end. The amplification product was gel-purified and cloned into *Hin*dIII and *Pst*I sites of pUC18 to yield construct pPFBVΔ3688–3923. Specific nucleotide substitutions were introduced in the viral cDNA of plasmid pSP18-IC with the Quick Change Site-Directed Mutagenesis kit (Stratagene) and proper oligonucleotide pairs to generate construct p37aug- and IRES mutants M1 to M10. The nucleotide sequence of all mutant constructs was verified with an ABI PRISM DNA sequencer 377 (Perkin-Elmer). The type and position of the modifications introduced in each construct have been indicated in the figures depicting the mutants.

Constructs embracing the sequences of PFBV sgRNAs were also generated. To this aim, the sequences of the 1.7 kb and 1.4 kb PFBV sgRNAs were PCR amplified from constructs pSP18-IC and pPFBVΔ2572–2648 using the Expand High Fidelity PCR System and primer CH52 (5′-GGTCTAGAGGGCGGGTTAAGGTCTCCATC-3′), complementary to the 3′ terminus of the viral sequence (nt 3903–3923) with a *Xba*I site (underlined) at the 5′ end, and either primer CH290 (5′-GGAAGCTTG*TAATACGACTCACTATAGGG*AAAGTCTGGCAGACCACACAATTG-3′), which contains a *Hin*dIII site (underlined) fused to a T7 RNA polymerase promoter sequence (in italics) followed by 21 nt of the 5′ end of the 1.7 kb sgRNA (positions 2262–2286 in the gRNA), or CH48 (5′-GAAGCTTG*TAATACGACTCACTATAGGG*ATAAACCTCCAACACATATTG), which contains 22 nt of the 5′ end of the 1.4 kb sgRNA (positions 2507–2528 in the gRNA). PCR products were run in 1% agarose gels, eluted, digested with *Hin*dIII and *Xba*I and subsequently cloned into the corresponding restriction sites of pUC18. Clones derived from pSP18-IC were named psg1.7 and psg1.4, respectively, and those derived from pPFBVΔ2572–2648 were named psg1.7Δ2572–2648 and psg1.4Δ2572–2648, respectively.

To obtain the bicistronic construct pH-L, the HIS3 and the lacZ genes were PCR amplified with the Expand High Fidelity PCR System from proper plasmids using specific oligonucleotides. The oligonucleotide homologous to the 5′- terminus of the HIS3 gene harbored a *Bam*HI site at the 5′-end, and the one complementary to the 3′-terminus contained a *Sph*I site at the 5′ end. The lacZ-specific oligonucleotides led to the incorporation of *Sph*I/*Bam*HI and *Pst*I sites at the 5′- and 3′-ends, respectively, of the corresponding PCR product. The amplified HIS3 and lacZ genes were ligated through the *Sph*I site and then cloned into *Bam*HI and *Pst*I sites of pBluescript KS (+) (Stratagene). Insertion of a 152 nt non-PFBV sequence or a PCR amplified segment of PFBV (nt 2505–2648) at the *Sph*I and *Bam*HI sites present between the HIS3 and lacZ genes of plasmid pH-L, gave rise to two new bicistronic constructs, pH-NV-L and pH-IRES-L, respectively. In order to fuse the expression cassette of the latter one (HIS-IRES-lacZ) to the UTRs of PFBV, a *Bam*HI site and a *Pst*I site were introduced with the Quick Change Site-Directed Mutagenesis kit into plasmid pSP18-IC before and after, respectively, the start and the termination codons of the p27 and the p37 genes to generate pSP18-BaPs. The DNA segment comprised between the *Bam*HI and *Pst*I sites of pSP18-BaPs was then replaced by the HIS-IRES-lacZ expression cassette to yield pH-IRES-L UTR.

Recombinant binary plasmids for agroinfiltration assays were obtained as follows. Restriction sites flanking p27 (*Spe*I and *Stu*I sites) and p37 (*Nco*I and *Pst*I sites) genes were consecutively introduced with the Quick Change Site-Directed Mutagenesis kit into pSP18-IC. These viral genes were exchanged, respectively, by the Rluc and the Fluc genes after their amplification with proper oligonucleotides from plasmids pBIP-RL [64] and RD29A::LUC [65], respectively. The resulting chimeric viral cDNA was PCR amplified, fused to the CaMV 35S promoter and the PoPit, and cloned into pMOG800 [66] to yield construct pV-RFF. An equivalent construct carrying an internal deletion in the viral cDNA, namely pΔV-RFF, was created by replacing a restriction fragment obtained by digestion with *Sal*I and *Nco*I by the corresponding one obtained from construct pPFBVΔ2505–2572.

### *In vitro* translation assays

Uncapped transcripts were synthesized *in vitro* with T7 RNA polymerase (Fermentas) from PFBV (genomic and subgenomic) constructs linearized with proper restriction enzymes. These transcripts were used for *in vitro* translation experiments with WGE Plus following manufacturer recommendations (Promega). [^14^C]Leucine was included in the reactions and the translation products were treated with 2% sodium dodecyl sulphate (SDS) and separated by electrophoresis through 7% to 15% polyacrylamide gels in Tris-glycine buffer [67]. The gels were subsequently fixed in 25% isopropanol and 10% acetic acid for 30 min, incubated with Amersham Amplify™ Fluorographic Reagent (GE Healthcare), dried and exposed to X-ray film or, alternatively, scanned with a bioimage analyzer (Fuji BAS1500) for quantitation of radioactive bands.

### Mechanical inoculation of plants and assessment of viral infection

Uncapped genomic PFBV RNA transcripts, synthesized as indicated above, were gently rubbed onto carborundum-dusted leaves of *C. quinoa* (three leaves per plant employing approximately 2 µg of transcript per leave). Plants were maintained under greenhouse conditions (16 h days at 24°C, 8 h nights at 20°C) and monitored for lesion appearance from 5 to 10 days post-inoculation (d.p.i). Leaf samples were harvested at different d.p.i. and infection was assessed by Northern blot hybridization with a ^32^P-labeled DNA probe derived from the 3′ terminal region of PFBV genome as previously described [27].

### *Agrobacterium*-mediated transient expression assay

Binary plasmids pV-RFF and pΔV-FF were introduced into *A. tumefaciens* strain C58C1 by heat shock. Transformed bacteria were grown overnight in proper conditions and subsequently collected by slow-speed centrifugation and adjusted to the required final OD_600_ (0.5) value with 10 mM MgCl_2_, 10 mM MES pH 5.6 and 150 µM acetosyringone. After 3 h incubation, the suspensions were used to infiltrate the abaxial side of *N. benthamiana* leaves using a 5 ml needleless syringe. The infiltrated plants were kept under greenhouse conditions and leaf samples were taken 3–4 days after infiltration. Leaf tissue was homogenized in the presence of passive lysis buffer (PLB, Promega) and the Rluc and Fluc luciferase activities were measured with the Dual-Luciferase Reporter Assay (Promega) employing a GloMax luminometer (Promega). The experiments were repeated more than three times for each construct.

### RNA secondary structure prediction

Secondary structure predictions were performed using the MFOLD program version 4.6 (www.bioinfo.rpi.edu/applications/mfold) [68], [69].
